# Basin Scale Soil Moisture Estimation with Grid SWAT and LESTKF Based on WSN

**DOI:** 10.3390/s24010035

**Published:** 2023-12-20

**Authors:** Ying Zhang, Jinliang Hou, Chunlin Huang

**Affiliations:** Key Laboratory of Remote Sensing of Gansu Province, Heihe Remote Sensing Experimental Research Station, Northwest Institute of Eco-Environment and Resources, Chinese Academy of Sciences, Lanzhou 730000, China; zhang_y@lzb.ac.cn (Y.Z.); jlhours@lzb.ac.cn (J.H.)

**Keywords:** soil moisture, WSN, LESTKF, SWAT

## Abstract

This research utilized in situ soil moisture observations in a coupled grid Soil and Water Assessment Tool (SWAT) and Parallel Data Assimilation Framework (PDAF) data assimilation system, resulting in significant enhancements in soil moisture estimation. By incorporating Wireless Sensor Network (WSN) data (WATERNET), the method captured and integrated local soil moisture characteristics, thereby improving regional model state estimations. The use of varying observation search radii with the Local Error-subspace Transform Kalman Filter (LESTKF) resulted in improved spatial and temporal assimilation performance, while also considering the impact of observation data uncertainties. The best performance (improvement of 0.006 m^3^/m^3^) of LESTKF was achieved with a 20 km observation search radii and 0.01 m^3^/m^3^ observation standard error. This study assimilated wireless sensor network data into a distributed model, presenting a departure from traditional methods. The high accuracy and resolution capabilities of WATERNET’s regional soil moisture observations were crucial, and its provision of multi-layered soil temperature and moisture observations presented new opportunities for integration into the data assimilation framework, further enhancing hydrological state estimations. This study’s implications are broad and relevant to regional-scale water resource research and management, particularly for freshwater resource scheduling at small basin scales.

## 1. Introduction

In watershed water resource management and land–atmosphere connections, soil moisture is a crucial factor that needs to be monitored [1,2]. For managers, basin-level observations are the most operational for scheduling and monitoring water resources effectively. There are two types of observations for soil moisture: remote sensing and in situ data. Remote sensing has been used for large-scale watershed water resource management and research for several decades, with a spatial resolution typically over 20 km [3]. However, this resolution is not as applicable to small basins. In recent years, new technologies have been developed to retrieve high-resolution soil moisture data from satellites. One such technology involves the merging of L-band radiometer retrievals and L-band radar observations to produce high-resolution soil moisture data based on the Soil Moisture Active and Passive (SMAP) mission, as developed by Das et al. [4]. Another approach is to combine the DISPATCH downscaling algorithm with CLASS to get a continuous series of soil moisture estimations at a 1 km resolution, as done by Djamai et al. [5]. High-resolution soil moisture data can be obtained through hydrological models, remote sensing and in situ data, scaling methods, or their combinations [6].

Various empirical soil moisture models, including the Kirchhoff Approximation Model (KAM) [7] and the Small Perturbation Model (SPM) [8], have been used to estimate soil moisture. These models were among the earliest approaches to analyzing scattering from rough surfaces, while more modern approaches like Integral Equation Model (IEM) [9] and Small Slope Approximation Method (SSAM) [10] are considered to have a larger validity range for monitoring scattering from rough surfaces. Shakya et al. [11] introduced a prototype of an IoT-based resistive soil moisture sensor, which offers voltage readings associated with varying levels of moisture in the soil. The main disadvantage of these models is that they follow a complex methodology and are quite challenging to implement. Physics-based hydrological models, such as VIC [12], LPJmL [13], and PCR-GLOBWB [14] can be used to estimate global or regional soil moisture through hydrologic processes modeling, which can be freely implemented by scholars with low physical exertion. Soil and Water Assessment Tool (SWAT) stands out as one of the most widely adopted hydrological models for representing the water cycle at the basin scale [15]. According to this model, the movement of soil water within the root zone can be described using a cascade model. Water is initially provided to the first layer after accounting for canopy interception and evaporation losses. Subsequently, based on factors such as field capacity and hydraulic conductivity, various processes occur, including storage, excess runoff, and infiltration. Any excess water that remains after these processes will then percolate into the subsequent layers. If the last layer becomes saturated and there is still excess water, the model redistributes it back to the first layer. Therefore, soil moisture [16], evapotranspiration [17], streamflow [18], sediment, nutrient components [19], and crop yield can be estimated with SWAT [20]. Its significance and utility in Earth physical studies and water resource management cannot be overstated [15]. With its versatility, scholars from diverse research interests have embraced SWAT as a highly valuable tool, transforming it into an echo-hydrological model enriched with cross-disciplinary knowledge. Notably, it has found extensive popularity in areas such as climate change research [21], Best Management Practices (BMPs) [22], water resource investigations [23], water quality assessment [24], and various scientific inquiries related to land surface hydrological processes [25], all conveniently facilitated by the capabilities of the SWAT model [26].

SWAT is typically implemented using specialized software such as the QSWAT 1.7 extension for QGIS 2.6.1 or the standalone SWAT Editor version 2012. These tools provide interfaces for model setup, input data management, and visualization of simulation results. Additionally, various pre- and post-processing tools are available to facilitate model calibration, sensitivity analysis, and result interpretation, such as SWAT-CUP [27]. There are some limitations in SWAT. The model requires extensive input data, which can be challenging to obtain and process. Model calibration and validation can be time-consuming and require significant effort. The model may require local calibration to accurately represent specific watershed characteristics. Uncertainties in input data and model assumptions can affect the accuracy of predictions. To ensure accurate estimations of soil moisture, it is crucial to calibrate and validate the model against observations, often relying on streamflow measurements. However, in situ soil moisture observations are seldom utilized for model calibration or constraint. This limitation hinders the model’s regional applications, highlighting the need for enhanced representation abilities.

**Figure 1:**
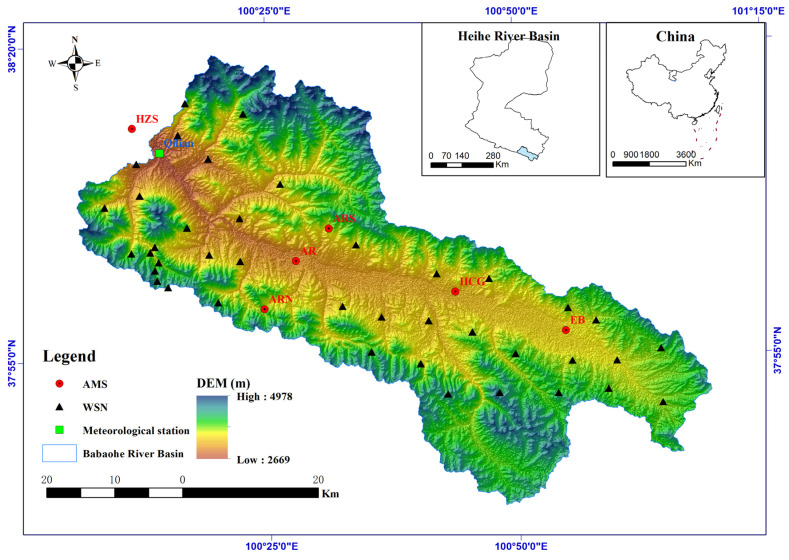
The location of Babaohe River basin.

The emergence of Wireless Sensor Network (WSN) technology has opened up new opportunities for data-intensive observations in ecological and hydrological research, as well as environmental monitoring. Initially, WSNs were deployed to compensate for the lack of space observation data in models used for research on ecological and hydrological processes in watersheds [28,29,30,31,32,33,34,35,36,37,38,39]. This limitation had previously hindered the accurate construction of modeling and simulation techniques [40]. A sub-basin of the Heihe River Basin called the Babao River Basin has been outfitted with WSNs for ecological and hydrological research [41]. This study takes the Babao River Basin as the research area, which is situated in the Qilian Mountain range and relies heavily on snow and ice melting for its water supply. To capture near-surface meteorological variation across different landscapes and elevation zones, six automatic meteorological stations (AMS) were installed (as shown in Figure 1) in the Babaohe river basin. Additionally, an optimal design based on the spatial variation of terrain, soil moisture, and soil temperature was used to deploy over 30 WATERNET nodes for a wireless sensor network [42]. This densely distributed network of automatic weather stations and WSN, complemented by the optimized observation sites, allows for the effective capture of major hydrological heterogeneity in the area. Although the WATERNET can provide high temporal resolution data on soil moisture conditions, its spatial observation range is still limited. Sparsely distributed sites cannot produce consistent soil moisture data across space. Consequently, this data is mainly used for model calibration or validation [43,44]. The lack of high-accuracy and high-resolution soil moisture data inhibits the precise representation of short-term or localized hydrological responses and meteorological variability across space and time.

The method of data assimilation is utilized to obtain soil moisture products that have high resolution and accuracy and are consistent in space and time [45]. This technology combines model estimates with observation data, applying Bayesian or optimal theory to enhance the accuracy of these estimates [46]. A novel approach by integrating in situ soil moisture observations into a coupled grid SWAT and PDAF data assimilation system was introduced in this study. Unlike traditional methods that mainly focus on satellite data downscaling, model calibration, and validation, our approach represents a significant departure by assimilating wireless sensor network data into a distributed model. This innovative strategy leverages the high accuracy and resolution capabilities of WATERNET’s regional soil moisture observations, providing valuable insights into local soil moisture characteristics and enabling the integration of multi-layered soil temperature and moisture observations into our data assimilation framework.

Hydrological studies usually involve the use of Kalman-type assimilation methods. The Singular Evolutive Interpolated Kalman (SEIK) filter has been found to be superior to the Ensemble Kalman Filter (EnKF) and the Singular Evolutive Extended Kalman (SEEK) filter [47]. To combine the best qualities of the ETKF and the SEIK filter, the Error Subspace Transform Kalman Filter (ESTKF) was developed. Specifically, the ESTKF can use a deterministic minimum transformation like the ETKF, while requiring lower computational resources. Unlike the SEIK filter, the ESTKF allows for the ensemble transformation to be independent of the order of the ensemble members in the ensemble matrix. Additionally, the ESTKF can utilize a random transformation similar to the SEIK filter, with the square root of the matrix computed via Cholesky decomposition, which is faster than the singular value decomposition needed for the minimum transformation. As a result, the ESTKF offers greater computational efficiency compared to the SEIK filter. Depending on the specific simulated problem, a unique technique using the Local Error-Subspace Transform Kalman Filter (LESTKF) with varying observation search radiuses to update local model states was implemented to improve spatial and temporal assimilation performance. Sea ice concentration and thickness derived from the satellites can be assimilated in the climate forecasting system using LESTKF to improve sea ice prediction skills [48,49]. If the localization radius is too large, spurious correlations in the background error covariance cannot be effectively filtered out. Conversely, if the localization radius is too small, the physical quantities field may not accurately reflect reality due to over-analysis [50]. Sobash and Stensrud [51] investigated how the EnKF’s sensitivity to the localization cutoff radius in the context of convective-scale data assimilation and perfect-model experiments. Their study revealed that enlarging the horizontal localization and reducing the vertical localization led to analyses with minimal errors in most state variables. Additionally, they delved into the influence of model error on determining the optimal localization radius. Trials with increased ensemble spread achieve lower RMSE values, indicating that variations in the ensemble spread may account for part of the sensitivity to the horizontal localization cutoff radius. As the localization cutoff radius decreases, resulting in fewer observations influencing the model state during the update at each grid point, the ensemble spread value increases [52]. This methodological innovation highlights the importance of carefully selecting an appropriate range for in situ soil moisture observations applications, which has not been extensively explored in previous studies.

The objectives of this study are as follows: (1) to evaluate the feasibility of estimating soil moisture in grid SWAT using sequential data assimilation of wireless sensor network in situ data, and (2) to analyze the impacts of observation search radius for data assimilation efficiency. In order to be able to validate the results, a synthetic study was designed. Assimilating real-world data was beyond this study’s scope. The validation data is required like high resolution soil moisture product. The structure of this paper is as follows: materials and methods (study area, WATERNET, GSWAT-PDAF, and LESTKF) are introduced in Section 2. The synthetic experiment setup is explained in Section 3. Section 4 presented the results and discussion. Section 5 concludes this research.

## 2. Materials and Methods

### 2.1. Study Area and Data Collection

The Qilian County is traversed by the Babao River, which is a tributary of the Heihe River. Spanning an area of 2455 km^2^, the Babao River has an elevation range of 2669 m to 4978 m above sea level (as illustrated in Figure 1). The mean annual precipitation exceeds 400mm, while evaporation rates are over 1500 mm. Alpine meadow, sub-alpine shrubbery, and alpine steppe are the dominant plant types in this region. The primary soil types include frigid desert soils, peat soil, typic alpine steppe soil, typic subalpine meadow soil, and alpine meadow soil, with the latter two being the most prevalent. To initiate the following grid SWAT project, a comprehensive data collection process was undertaken. All necessary input data were sourced from publicly available online resources (Table 1), including weather station data, Digital Elevation Model (DEM) data from SRTM—Shuttle Radar Topography Mission [53], soil type map derived from HWSD v1.2—Harmonized World Soil Database v1.2 [54], and land use map derived from MCD12Q1—MODIS/Terra+Aqua Land Cover Type Yearly L3 Global 500 m SIN Grid [55]. Specifically, climate input data was extracted from six automatic meteorological stations (AMS) derived from HiWATER [41] and utilized as the primary weather generator for grid SWAT.

### 2.2. Grid SWAT Model

The Soil and Water Assessment Tool (SWAT) is a widely used semi-distributed eco-hydrological model employed in water resource management. In our study, we extended the capabilities of SWAT by gridding it at the Hydrologic Response Unit (HRU) level. The gridding method employed in the project involved setting the spatial resolution at 500 m × 500 m due to the highest resolution of the input parameters being 500 m × 500 m. This approach enabled the Babaohe river basin to be divided into 9707 HRUs. To facilitate the building of the Babaohe river basin SWAT project, the ArcSWAT version 2012 extension of ArcGIS 10.7 software was utilized.

To ensure the specific requirements of the application were met, the configuration and control files for the GSWAT model were meticulously prepared. The time step for the simulation was set at a daily frequency. By following this well-defined and systematically planned workflow, the gridded SWAT project was effectively established, providing accurate and reliable ecohydrological data for analysis.

GSWAT parameters were calibrated using streamflow observations from the Qilian gauge station. The calibration process relied on a Latin-hypercube one-factor-at-a-time (LH-OAT) sensitivity analysis and particle swarm optimization method. Subsequently, the parameters were validated for the Babaohe River Basin. Based on sensitivity analysis, nine highly sensitive parameters (ALPHA_BF, CH_K2, CN2, SOL_AWC, ESCO, CH_N2, SFTMP, and SURLAG) were calibrated for the period of 2000–2004 and validated for the period of 2005–2007, as shown in Table 2.

### 2.3. WATERNET Data

WATERNET is a wireless sensor network designed to collect in situ soil moisture observations. These observation sites utilize wireless transmission techniques to communicate with the central data center [56]. In this dataset, 4 cm, 10 cm, and 20 cm soil moisture are the basic observations for each node. There are 19 nodes that include observations of soil moisture and surface infrared radiation temperature. Additionally, there are 11 nodes that include observations of soil moisture, surface infrared radiation temperature, snow depth, and precipitation. The observation frequency is every 5 min. The implementation of WATERNET has been successfully carried out in the Heihe River Basin [41]. It has proven effective in achieving two primary objectives: capturing the spatial variations and temporal dynamics of soil moisture and soil temperature at multiple scales, and providing ground truth estimates for remote sensing with an approximate kilometer pixel scale by employing spatial upscaling techniques. This dataset can be accessed from A Big Earth Data Platform for Three Poles (http://poles.tpdc.ac.cn/ (accessed on 1 January 2023)) [57].

In the midstream areas of the Heihe River Basin, where crops constitute the dominant vegetation type and human-induced irrigations are prevalent, the representation of soil moisture by WATERNET may have certain limitations [58]. Conversely, in the upstream region of the Heihe River Basin, which is primarily a natural ecosystem, WATERNET installations play a crucial role. When deploying WATERNET in a river basin, the selection of optimal observation locations becomes a critical initial step. These observation sites should be strategically placed to capture the spatial variation and distribution of ecohydrological variables.

**Figure 2:**
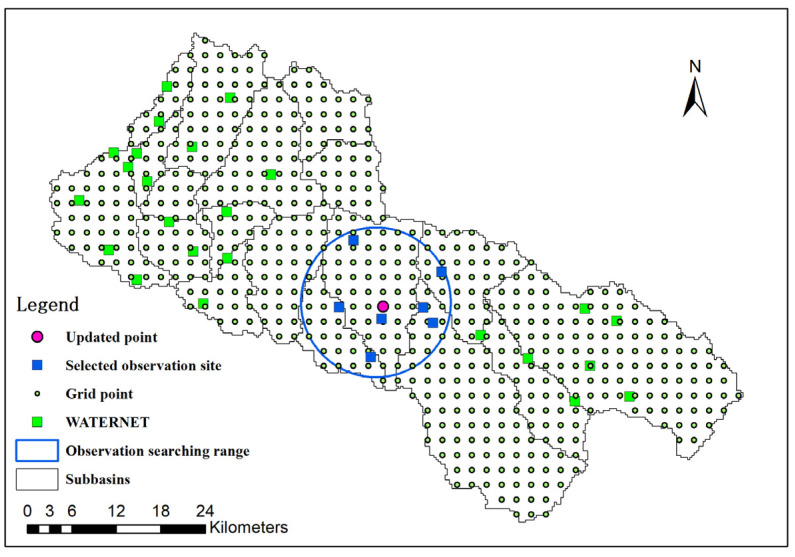
Observation searching range (blue circle) for a single grid (purple point) in LESTKF. Green box is WATERNET observation site. Blue box is observation used for updating purple point state.

Ge et al. [42] proposed a geostatistical method for optimizing the design of multivariate sampling in WATERNET installations. This method utilizes a universal cokriging model to ensure efficient capture of the spatial variation in the target variables and to monitor ecohydrological processes in the Babaohe River Basin. Currently, in the upstream Babaohe River Basin, 32 WATERNET sites have been consistently providing multi-layer soil moisture data for users, as depicted in Figure 2.

### 2.4. Ensemble Kalman Filter

The Ensemble Kalman Filter (EnKF) data assimilation method was utilized to compare with LESTKF method in this study. The formalization of the EnKF involves producing model error estimates through the assumption that the ensemble mean represents the “truth” and calculating the variance of the differences between each ensemble member and the ensemble mean. Subsequently, every individual observation is updated based on the relative error found in both the model and observations. The EnKF can be formalized as follows.

(1) $$X_{i,0}^{a}=X_{0}^{a}+u_{i},\;\;u_{i}\sim N(0,P_{0})$$

where $X_{0}^{a}$ is initial state, $X_{i,0}^{a}$ is the state of the *i*-th element. $u_{i}$ is the background error

(2) $$X_{i,k+1}^{f}=M\left(X_{i,k}^{a},\alpha_{k+1},\beta_{k+1}\right)+w_{i},\;\;w_{i}\sim N(0,Q)$$

where *M*(·) is the model operator (GSWAT), a and f present the analysis and forecast values of the state variables (4 cm depth soil moisture), and $x_{i,k+1}^{f}$ is the *i*-th element of the state forecast set at time *k* + 1 which is driven by two fundamental factors: meteorological data and model parameters, denoted as alpha and beta, respectively. It can be seen that land surface variables calculated based on process-oriented models are highly sensitive to spatio-temporal variations in surface heterogeneity and meteorological conditions. The model error term $w_{i}$ follows a Gaussian distribution with mean 0 and variance Q, representing the uncertainty related to model parameters and structure.

When observation data are available, the forecast state variables are updated using the following linear formula:

(3) $$X_{i,k+1}^{a}=X_{i,k+1}^{f}+K_{k+1}(Y_{i,k+1}-{\overset{\wedge }{Y}}_{i,k+1})$$

(4) $${\overset{\wedge }{Y}}_{i,k+1}=H(X_{i,k+1}^{f})$$

Here, $Y_{i,k+1}$ is the perturbed observation set obtained by adding noise to the original observation data, which follows a Gaussian distribution with mean 0 and variance *R*. Generally, it is assumed that the observation error matrix *R* is diagonal, indicating that multiple observation variables are independent of each other and stable over time. ${\overset{\wedge }{Y}}_{i,k+1}$ is the projection of the model state variable onto the observation space, and *H*(·) is the observation operator used to establish the relationship between the model state variable and the observation data, which can be either linear or nonlinear. *K_k_*_+1_ is the Kalman gain matrix at time *k* + 1, calculated as follows:

(5) $$K_{k+1}=P_{k+1}^{f}H^{T}(HP_{k+1}^{f}H^{T}+R{)}^{-1}$$

Here, $P_{k+1}^{f}$ is the forecast error covariance matrix at time *k* + 1, $P_{k+1}^{f}H^{T}$ is the cross-covariance matrix between the model state variable $x_{k+1}^{f}$ and its projection onto the observation space $H(x_{k+1}^{f})$, and $HP_{k+1}^{f}H^{T}$ is the error covariance matrix of $H(x_{k+1}^{f})$. The analysis value of the state variable at time *k* + 1 is given by the ensemble mean, and the updated analysis set is returned to the model for the next forecast. When new observation data become available, the above process is repeated for state updating.

(6) $$P_{k+1}^{f}=\frac{1}{N-1}\sum_{i=1}^{N}(X_{i,k+1}^{f}-\bar{X_{k+1}^{f}})(X_{i,k+1}^{f}-\bar{X_{k+1}^{f}}{)}^{T}$$

(7) $$P_{k+1}^{f}H^{T}=\frac{1}{N-1}\sum_{i=1}^{N}(X_{i,k+1}^{f}-\bar{X_{k+1}^{f}})[H(X_{i,k+1}^{f})-H(\bar{X_{i,k+1}^{f}}){]}^{T}$$

(8) $$HP_{k+1}^{f}H^{T}=\frac{1}{N-1}\sum_{i=1}^{N}[H(X_{i,k+1}^{f})-H(\bar{X_{i,k+1}^{f}})][H(X_{i,k+1}^{f})-H(\bar{X_{i,k+1}^{f}}){]}^{T}$$

Here, the overline above the expression denotes the mean value of the corresponding variable. The analysis value of the state variables (surface soil moisture) at time *k* + 1 is given by the ensemble mean. The updated analysis ensemble then returns to influence the model for the next forecast. When the next set of observation data appears, the above process is repeated for state updating.

### 2.5. Local Error Subspace Transform Kalman Filter

This localized variant of the Error-Subspace Transform Kalman Filter is a sequential data assimilation method, which follows a systematic workflow. As the system runs, observations become available, and these are used along with model error to update the model state, resulting in an optimized model state. The updated model state is then used to reinitialize and propagate until a new observation becomes available. In order to improve model state estimation based on nearby relevant observations, localization for the filter or interpolation for data can be applied.

In our study, we found that a model state can be effectively updated using observation data within an observation searching circle (as illustrated in Figure 2). Since the in situ soil moisture data we used in our study typically represented small-scale features, it was essential to implement a local filter to accurately capture these features. Moreover, the quantity of observations also played a critical role in determining the optimal approach. To this end, we set six different search radii for LESTKF to examine the observation representation range and to determine the most effective strategy for achieving accurate and reliable model state estimations.

In all ensemble-based Kalman filters, the state vector y of size m and the corresponding error covariance matrix $P^{y}$ represent the state of a physical system and its error estimate at time *t*. These quantities are represented by an ensemble of m vectors $Y_{k}$ of model state realizations. The state (4 cm depth soil moisture) estimate is given by the ensemble mean. The analysis of ESTKF at time t can be constructed as follows:

(9) $$Y_{k}=[y_{1},\;\ldots ,y_{k}];\;k=1,\ldots m$$

(10) $$Y^{f}=M\;[Y]\;$$

where *M* is model operator. $Y^{f}$ is forecasted state vector at time *t* + 1.

(11) $$Y^{\prime }=Y-\bar{Y}$$

(12) $$P^{y}=\frac{1}{k-1}Y^{\prime }(Y^{\prime }{)}^{T}$$

For the ESTKF, the forecast covariance matrix $P_{}^{f}$ is written as

(13) $$P_{}^{f}=\frac{1}{k-1}F(F{)}^{T}$$

where *F* is a matrix of size *n* × (*m* − 1) defined by

(14) $$F=Y_{}^{f}T$$

(15) $$T_{i,j}=\left\{\begin{array}{l} 1-\frac{1}{m}\frac{1}{\frac{1}{\sqrt{m}}+1}\;for\;i=j,\;j<m,\\ -\frac{1}{m}\frac{1}{\frac{1}{\sqrt{m}}+1}\;for\;i\neq j,\;j<m,\\ -\frac{1}{\sqrt{m}}\;for\;j=m. \end{array}\right.$$

*T* is the Householder matrix associated with the vector *m*^−1/2^(1,…,1)*^T^*. *i* is the column number and *j* is the row number.

For the analysis, one defines a transform matrix *S* of size (*m* − 1) × (*m* − 1) by

(16) $$S^{-1}=\rho (m-1)I+(HF{)}^{T}R^{-1}HF$$

where *I* is the identity and ρ with 0 < ρ ≤ 1 is the ‘forgetting factor’ that is used to implicitly inflate the forecast error covariance estimate. *H* is the measurement operator. The observation vector *Z* of size *q* is related to the model state by *Z* = *H* ($Y_{}^{f}$) + *σ*. The vector of observation errors *σ* is assumed to be a white Gaussian distributed random process with covariance matrix *R*. The analysis covariance matrix is defined as

(17) $$P_{}^{a}=FS(F{)}^{T}.$$

The analysis ensemble is computed as a correction of the ensemble mean and a transformation of the ensemble perturbations. The analysis state estimation is calculated from the forecast using the combination of the columns of the matrix *F* by

(18) $$\bar{y_{}^{a}}=\bar{y_{}^{f}}+F\bar{w},$$

with the weight vector $\bar{w}$ of size *m* − 1 given by

(19) $$\bar{w}=S(HF)TR(z_{i}-H\bar{y}).$$

The ensemble is now transformed as

(20) $$y^{a}=\bar{y_{}^{a}}+FW$$

where the weight matrix *W* is calculated as

(21) $$W=\sqrt{m-1}CT^{T}\Lambda .$$

Here, *C* is the symmetric square root of *S* that is computed from the singular value decomposition UAV = *S*^−1^ such that *C* = UA^−1/2^U*^T^*. The matrix Λ of size *m* × *m* is an arbitrary orthogonal matrix or the identity. The vector (1,…,1)*^T^* has to be an eigenvector of Λ to ensure that the ensemble mean is preserved.

The analysis state estimation can be combined as

(22) $$Y^{a}=\bar{Y_{}^{f}}+F(\bar{W}+W)$$

where $\bar{W}$ = [$\bar{w}$,…, $\bar{w}$].

To perform local analysis, the study calculates the local analysis error covariance and perturbations in the ensemble space for each model grid cell using selected local observations. These observations are weighted based on their distance from the target domain by element-wise multiplication of the inverse matrix *R* with a localization matrix *L*. The localization matrix *L* is constructed using a correlation function that has compact support, ensuring that observations beyond a certain distance from the target domain are given zero weight. This approach effectively limits the influence of distant observations on the local analysis. The analysis of LESTKF can be described as

(23) $$Y_{L}^{a}=\bar{Y_{L}^{f}}+F_{L}({\bar{W}}_{L}+W_{L})$$

The LESTKF scheme involves a weighting function for localization achieved by dividing the area into local patches. This function assigns a weight of 1 within the patch and 0 outside it, and scales the observational error covariance based on the distance from the center of the local patch. The covariance localization via the weighting function within the local patch works by assigning larger errors to more distant observations. The localization of covariance through the weighting function within the local patch operates by assigning greater errors to observations that are farther away. When the weighting function of covariance localization is centered closely around the local patch center, the scheme exhibits similarities to a 1 − D filter. This resemblance can be achieved by multiplying the observation error covariance by the inverse of the smooth weighting function within each local patch. The weighting function typically ranges from 0 to 1.

Hydrologic model states such as the 0−4 cm layer soil moisture are initialized with an initial state (e.g., soil moisture and snow water equivalent) on 1 April. The prediction step evolves the hydrologic model’s initial states until a new WATERNET soil moisture observation within its searching radii becomes available, and the analysis step is triggered. In the analysis step, we use LESTK to update the estimated soil moisture ($Y_{L}^{f}$) by perturbating soil moisture observation from WATERNET and estimated soil moisture variance corresponding to observations and model soil moisture. After that, new initial conditions (updated state, $Y_{L}^{a}$) are evolved in the hydrologic model.

### 2.6. GSWAT-PDAF Data Assimilation System

**Figure 3:**
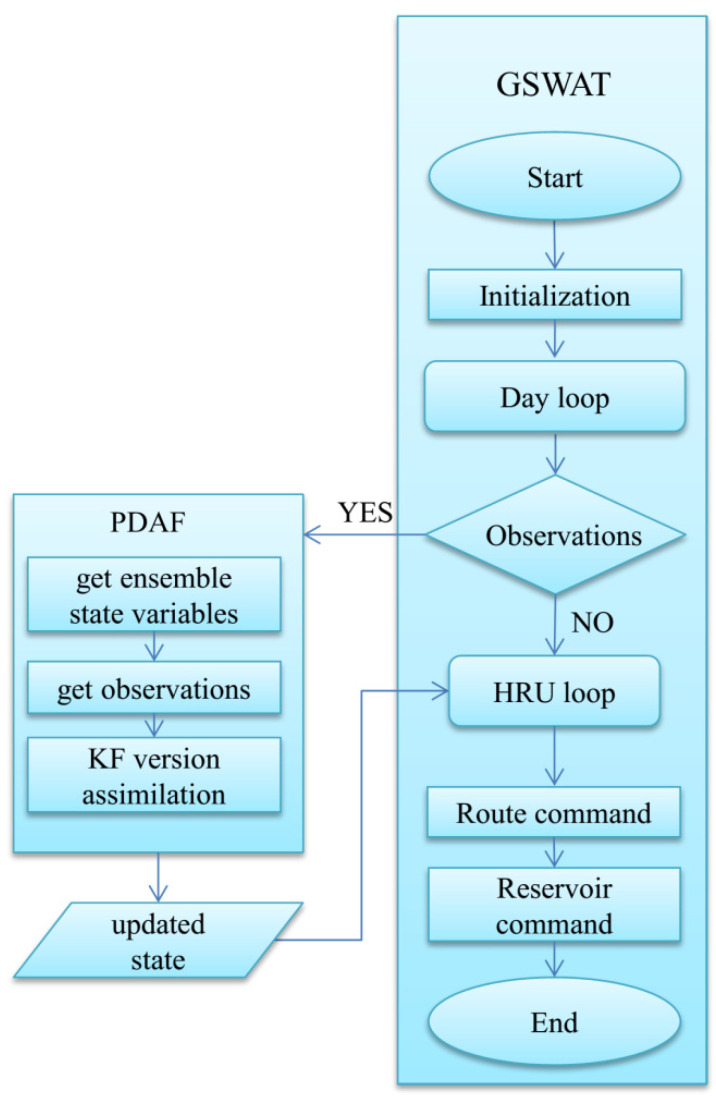
The coupling structure of GSWAT-PDAF.

In our study, we integrated Grid SWAT (GSWAT) with the Parallel Data Assimilation Framework (PDAF), as shown in Figure 3. PDAF encompasses various data assimilation methods [59]. By gridding SWAT at the HRU level, each grid cell has a unique spatial location and uniform size, facilitating the integration of grid data and the model itself. The inputs for the gridded SWAT (referred to as GSWAT) include precipitation, air temperature, humidity, wind, and solar radiation, among others. PDAF was modified to act as an interface for GSWAT, and the coupling between the two models is achieved through the exchange of input/output files in offline mode [60].

The workflow of the coupled GSWAT-PDAF system can be described as follows: First, the model state and observation data are outputted to files and subsequently read by PDAF. Then, using the LESTKF method, PDAF updates the model state based on the observation data and outputs the analyzed state data. Finally, the model reads the analyzed state data as the initial conditions for the next step in the simulation. This integrated approach allows for improved data assimilation and enhanced estimation of hydrological states within the GSWAT framework.

## 3. Experiment Setup

### 3.1. Observing System Simulation Experiments (OSSEs)

**Figure 4:**
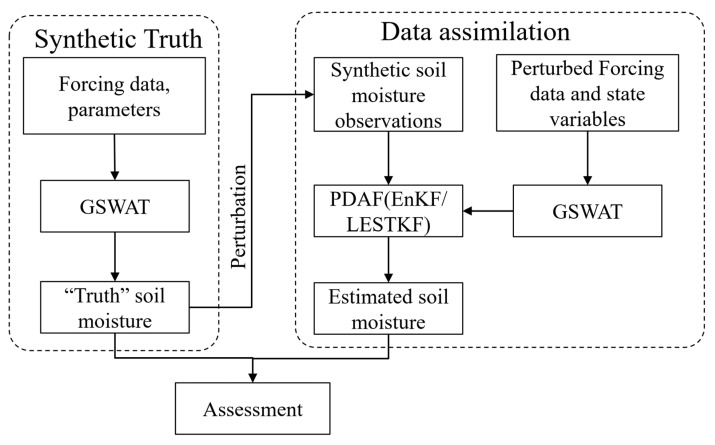
The flowchart of the study.

OSSEs are employed to validate the data assimilation method under ideal conditions, assuming that the system’s state and the error statistics of both the model and observations are accurately known. In these OSSEs, simulated data act as virtual observations for the data assimilation experiments. We carried out OSSEs for the Babaohe River Basin using GSWAT-PDAF to evaluate the performance of the LESTKF and EnKF separately in this particular study. We assimilated synthetic 4 cm depth soil moisture observations into the system to estimate the 4 cm depth soil moisture of the watershed. These sets of synthetic experiments aim to illustrate the impact of uncertainties in forcing input and physical representations on the assimilation process’s sensitivity. The flowchart of the study was illustrated in Figure 4.

### 3.2. Benchmark and Ensemble Generation

In this section, the study conducted a soil moisture data assimilation experiment using wireless sensor networks (WSN) and the GSWAT-PDAF model with LESTKF and EnKF separately under different spatial settings (Figure 4). The basic structure of a synthetic “twin” experiment is as follows. A control run was performed for the period from April 1st to May 1st in 2008 after a year of spin-up, using true soil, vegetation, and forcing inputs which served as a baseline for comparison with data assimilation and open loop simulations from the coupled GSWAT-PDAF framework with and without LESTKF/EnKF updating. The surface soil moisture data were outputted as the “truth” for evaluation purposes. The uncertainties in the model predictions can be mainly attributed to forcing input errors; therefore, we utilize perturbed forcing data to generate the ensemble members to include such uncertainties. The ensemble simulations for GSWAT-PDAF are performed in an open-loop experiment from 1 April to 1 May in 2008 and are driven by different forcing data sets (i.e., the precipitation data multiplied by normally distributed random noise). In the data assimilation case, the synthetic data are assimilated into the GSWAT-PDAF from 1 April to 1 May in 2008 and are driven by the same forcing data sets as the open-loop experiment.

For both the open-loop and data assimilation experiments, the generation of ensembles is carried out by introducing perturbations to the forcing input and model states. For air temperature, perturbations were added using an additive approach with a standard deviation of 1K. As for precipitation data (*Pr*), lognormally distributed noise was applied by multiplying it with a standard deviation of $\sigma_{p}=\sqrt{ln\left({\left(0.5*Pr\right)}^{2}/{Pr}^{2}+1\right)}$. To introduce model errors, the precipitation amount was multiplied by a scale factor of 1.5. Additionally, the daily cycle of the air temperature data was expanded to a range of [Tmin − 2K, Tmax + 2K], where Tmin and Tmax represent the minimum and maximum daily air temperatures, respectively. These perturbed forcing data were utilized for both data assimilation and open loop runs. The state variable (surface soil moisture) of the model was also perturbed with an additive error with 0.005 m^3^/m^3^ standard deviation. For this research experiment, a total of 20 ensemble members are utilized on a high-end server.

### 3.3. Soil Moisture Data Assimilation Experiment

**Figure 5:**
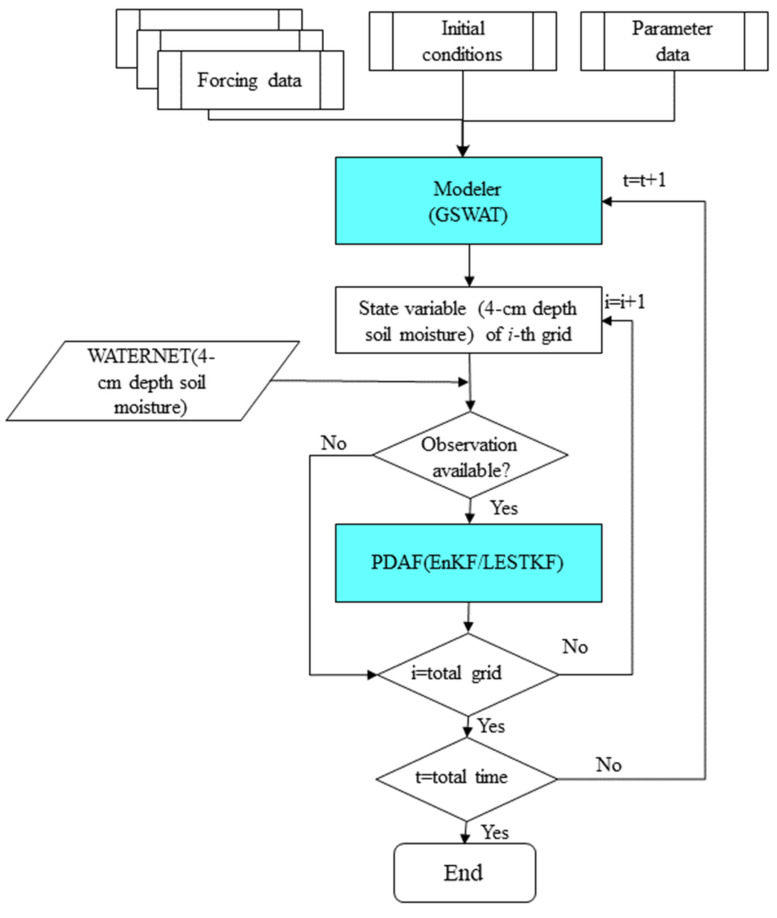
Flowchart of the soil moisture data assimilation with EnKF/LESTKF using GSWAT-PDAF.

In this section, a suite of experiments was performed to assimilate surface soil moisture data into GSWAT-PDAF with LESTKF and EnKF. Three synthetic 4 cm depth soil moisture observation datasets were generated by introducing Gaussian distributed errors with a standard deviation of 0.01 m^3^/m^3^, 0.03 m^3^/m^3^, and 0.05 m^3^/m^3^ into the truth separately to mimic the observation uncertainties. The standard deviations of the 4 cm depth soil moisture observations are based on the data derived from in situ data (to ensure the realism of the experiment). The observations had the same spatial distribution as WATERNET. The observation search radii for LESTKF were set at 0 km, 5 km, 10 km, 20 km, 40 km, and 50 km. The state variable (4 cm depth soil moisture) is only updated with EnKF/LESTKF when the observation located within its search scope. The flowchart of the WATERNET soil moisture observations data assimilation with LESTKF/EnKF using GSWAT-PDAF is shown in Figure 5.

## 4. Results and Discussion

### 4.1. Performance of the Soil Moisture Data Assimilation Experiment

Spatial and temporal averaged Root-Mean-Square Errors (RMSEs) for soil moisture estimation from open loop and assimilation scenarios were examined. $Qo_{t}$ is observed surface soil moisture at time *t* for all grid cell, *M* is the maximum time steps, $\bar{Qs_{t}}$ is the ensemble mean simulated state variable at time *t* for all grid cells. The temporal average RMSE is calculated as

(24) $$\mathrm{RMSE}=\sqrt{\frac{1}{M}\sum_{t=1}^{M}(\bar{Qs_{t}}-Qo_{t}{)}^{2}}.$$

$Qo_{i}$ is observed surface soil moisture at grid cell *i* for the whole time, *N* is the maximum grid cell, $\bar{Qs_{i}}$ is the ensemble mean simulated state variable at grid cell *i* for the whole time. The spatial average RMSE is calculated as

(25) $$\mathrm{RMSE}=\sqrt{\frac{1}{N}\sum_{i=1}^{N}(\bar{Qs_{i}}-Qo_{i}{)}^{2}}.$$

The percentage bias (*Pbias*) was calculated and defined as follows:

(26) $$Pbias=\frac{\sqrt{\sum_{t=1}^{M}\sum_{i=1}^{N}\left(\bar{Qs_{i,t}}-Qo_{i,t}\right)}}{\sqrt{\sum_{t=1}^{M}\sum_{i=1}^{N}Qo_{i,t}}}\times 100$$

**Figure 6:**
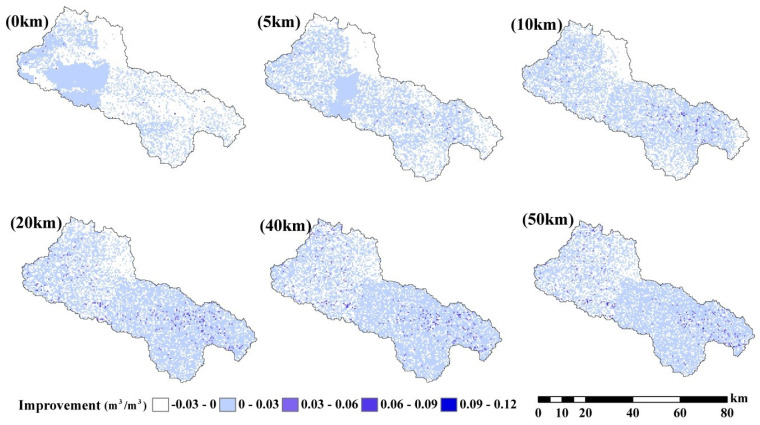
Surface soil moisture estimation improvement (RMSEop−RMSEda) by assimilation WATERNET data with 0.03 m^3^/m^3^ standard error based on LESTKF with (0 km, 5 km, 10 km, 20 km, 40 km and 50 km) observation search radius.

This study evaluated the performance improvements (measured as the difference between open-loop RMSE (RMSEop) and assimilation RMSE (RMSEda)) for various soil moisture data assimilation scenarios (as shown in Figure 6). Notably, there was a clear enhancement in soil moisture estimations near the WATERNET locations. Furthermore, with a search radius of less than 40 km, increasing the radius resulted in a spatial distribution of improvements that tended to be more steady and cover a wider area of the basin. The patterns of improvement performance were similar from 40 km to 50 km. However, for a search radius larger than 40 km, further enlargement of the radius did not result in an expansion of the improvement regions.

The areas showing the greatest improvement were concentrated along the flat plain in the east upstream region, while the downstream areas exhibited the least improvement, particularly when the search radius was short. This observation suggests that WATERNET observations from the upstream regions are more informative for soil moisture estimation in nearby areas. Interestingly, the density of WATERNET did not significantly affect the distribution of improvement. The distribution of improvement closely matches the topography, as shown in Figure 1, indicating that topography plays a significant role in soil moisture observations [61]. In areas with flat ground topography, soil moisture observations from those sites can effectively represent the soil moisture characteristics of most surrounding areas. However, in downstream regions with complex terrain, the in situ observations may not accurately represent the soil moisture conditions in that area.

**Figure 7:**
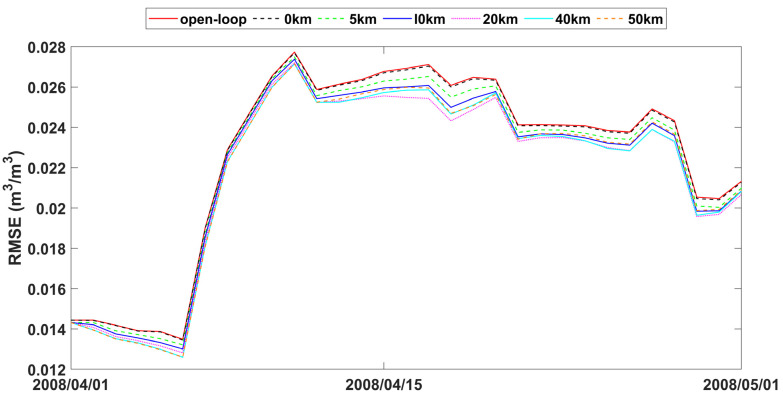
RMSE time series of spatial averaged soil moisture from 1 April to 1 May 2008 for open-loop and LESTKF assimilation scenario with local search range from 0 km to 50 km and 0.01 m^3^/m^3^ observation standard error.

**Figure 8:**
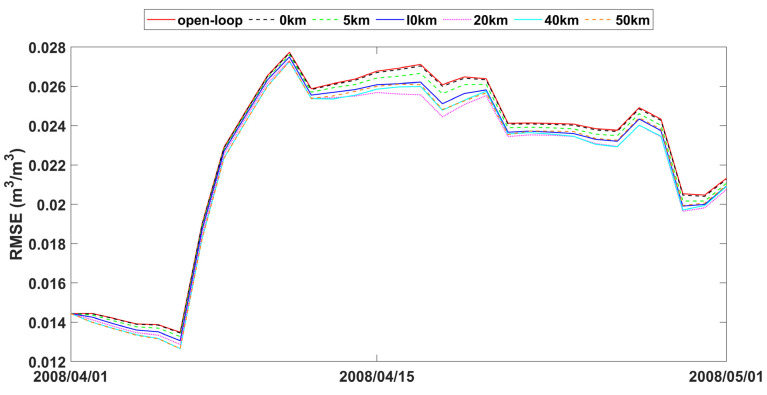
RMSE time series of spatial averaged soil moisture from 1 April to 1 May 2008 for open-loop and LESTKF assimilation scenario with local search range from 0 km to 50 km and 0.03 m^3^/m^3^ observation standard error.

**Figure 9:**
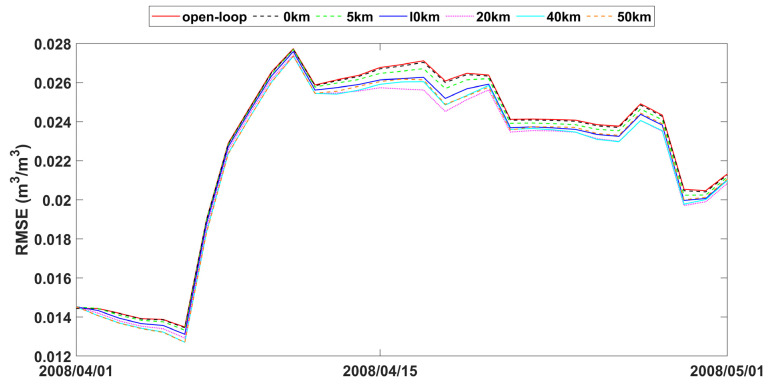
RMSE time series of spatial averaged soil moisture from 1 April to 1 May 2008 for open-loop and LESTKF assimilation scenario with local search range from 0 km to 50 km and 0.05 m^3^/m^3^ observation standard error.

The study also analyzed time series data of spatially averaged RMSEs from open-loop and assimilation scenarios with varying observation search radii, as presented in Figure 7, Figure 8 and Figure 9. The fluctuations in error correction were consistent across the different search radii. Specifically, when the search radius was between 0 km and 20 km, the error correction capacity of LESKTF increased with an increase in the search radius. However, when the radius exceeds 20 km, the error correction became ambiguous and did not show a clear trend.

The statistics of improvement based on LESTKF with different observation search radiuses was calculated based on the difference between the RMSE result of open-loop experiment and the RMSE result of LESTKF with different observation search radiuses. The greater the difference, the better the performance of the LESTKF (Figure 7, Figure 8 and Figure 9). As shown in Table 3, the improvement in data assimilation increases with an increase in observation search radius. The optimal performance of data assimilation is achieved when the observation search radius exceeds 20 km. However, enlarging the search radius beyond 20 km do not yield significant improvements. It is important to note that there exists a certain applicable range for WATERNET observations, such as the 20 km range used in this study.

The performance of soil moisture in LESTKF is better than EnKF during open-loop periods (Table 4). The *Pbias* and RMSE of LESTKF are lower than EnKF and open loop. The *Pbias* and RMSE of EnKF are nearly the same as open loop indicating that EnKF nearly did not work effectively. The time cost for the LESTKF is also less than that for the EnKF. With sparse observations, LESTKF is more useful and efficient than EnKF. With the different observation standard errors, LESTKF performed differently. The lower the observation standard error is, the better the performance of LESTKF is. The best performance of soil moisture estimation is the LESTKF scenario with 0.01 m^3^/m^3^ observation standard error. High accuracy soil moisture observations from wireless sensor networks can improve the LESTKF data assimilation performance. Consequently, the deployment of wireless sensors, such as WATERNET, is very useful for regional soil moisture estimation or other hydrological predictions. In general, the localized formulation of the Error-Subspace Transform Kalman Filter (LESTKF) outperforms the ensemble square root filters (EnSRF) [62]. When using localized EnSRF, it is crucial to set the observation localization radius within a reasonable range [63]. For instance, the radius can be larger for flat plain areas compared to complex terrains. Conducting a synthetic experiment becomes necessary in order to determine the appropriate radius for practical data assimilation usage.

### 4.2. Comparisons with Relevant Literatures

Localization not only improves accuracy but also reduces computational costs by utilizing only a portion of the complete state in the filter [64]. Han et al. [65] used the different localization length which was estimated at each assimilation step and their results showed that incorporating additional local brightness temperature data will enhance the estimation of model grid cells lacking observation data, with a maximum limit of nine local observations in this particular scenario. The distributed hydrological model effectively assimilated multiple remotely sensed soil moisture values for each grid through the use of observation operators and geolocation-based observation localization, which facilitated consistent updates of soil moisture within the model [66]. Li et al. [67] found that as the localization length scale increases, the biases of the merged soil moisture decrease. The biases reach their minimum value when the length scale is set to 100 km when they assimilated in situ soil moisture observations into land surface models with localization length scales of 10 km, 30 km, 50 km, 80 km, 100 km, 150 km, and 200 km. The above results are similar to the optimal search range of 20 km in our study. However, they did not compare their results with other data assimilation methods such as EnKF and assess the effect of the observation uncertainties on data assimilation.

Similar localization techniques have been documented in earth system data assimilation such as discharge [68], carbon [69], sea surface temperature [70], and sea ice concertation [48,71]. Additional approaches to address potential spurious correlations between variables include adaptive localization [72] and the use of two iterative filters instead of one [73]. The adaptive localization-based filter can improve the accuracy as well as the convergence of ensemble-based methods in the context of sequential data assimilation methods [74,75,76]. The degree of skill improvement also varies significantly by region which were attributed to the number of assimilated observations [77]. In another hand, larger localization does not necessarily work uniformly. For example, improving the representation of the smaller scales in the analysis by using a smaller localization radius has a positive effect on the larger scales, even though the analysis sensitivity to localization on those scales is low [52]. Localization is an essential element of ensemble-based Kalman filters in large-scale systems [63]. This study indicated that the localization configuration is necessary and should also be optimized in small-scale systems (regional water cycle system).

Observation errors encompass measurement errors, representativeness errors, and errors arising from inaccuracies in the observation operator. Estimating these errors accurately is a challenging task. Therefore, modern Observing System Simulation Experiments (OSSEs) necessitate the calibration of error settings to ensure that they can provide impact assessments similar to those obtained from Observing System Experiments (OSEs) using the same Observation Data Assimilation and Prediction (ODAP) systems. This calibration can be crucial for generating consistent results even when assimilating real observations. The requirements for modern OSSEs are outlined in Hoffman and Atlas [78]. The spatial correlation in the observation errors cannot be neglected in some cases, for example, Waller et al. [79] estimated correlation length scale of 7 km is longer than the expected value of 250 m. The WATERNET observations used in this study are sparsely distributed; therefore, the spatial correlation in the observation can be neglected in this study. Utilizing precise estimates of observation error statistics can aid in implementing quality control protocols and determining the optimal observations to assimilate for maximum benefit.

### 4.3. Limitations and Prospects of This Study

Limitations of the study include:

(1) Data source bias: The results of the hydrological data assimilation may be influenced by bias in the data source. The forcing data (e.g., precipitation and air temperature) used comes from reanalysis data or a limited number of monitoring stations and these stations have spatial distribution biases, then the assimilation results may be affected by this spatial bias. Then the high spatiotemporal resolution and accuracy forcing data is needed in the hydrological data assimilation applications.
(2) Model structure bias: The structure and parameter selection of the hydrological model (Grid SWAT model) itself may lead to bias in the assimilation results. When the model structure is not accurate enough or the parameter settings are unreasonable, it may affect the quality of the assimilation results.
(3) Measurement errors and uncertainty: Measurement errors and uncertainty in WATERNET observation data are important limiting factors in this hydrological data assimilation. If the precision of observation data is not high or there is significant uncertainty, the accuracy and reliability of the assimilation results may be affected.
(4) Temporal and spatial resolution: The precision and credibility of hydrological data assimilation results are also related to the temporal and spatial resolution of observation data. If the temporal and spatial resolution of observation data is insufficient to capture the details of surface hydrological processes, then the assimilation results may be limited.
(5) Prior information: The prior information, including the initial state of the system, such as soil moisture content, groundwater levels, or snowpack conditions, used in hydrological data assimilation may also introduce bias. Prior information helps to constrain the range of possible solutions during data assimilation, providing a starting point for estimating the current state of the system. It is used in combination with observation data to improve the accuracy and reliability of the assimilated results. If the prior information is inaccurate or incomplete, the assimilation results may be affected by this prior information.

Possible future improvement of the study can include:

(1) Inflation factor: As a result of unaccounted model errors and a restricted ensemble size, state and parameter uncertainties may decrease to an insufficient level during assimilation [80]. The primary challenge in practical applications lies in accurately representing model uncertainties to prevent the emergence of spurious covariance during data assimilation. While assimilating observations, the uncertainty in parameters and states gradually decreased over time. However, despite this reduction in uncertainty, incorrect updates of parameters and states were obtained. These errors could not be rectified by assimilating additional observations to improve the representation of the hydrological system. Inflation methods can effectively increase state uncertainties. Along with the localization method, the inflation factors can also be an improvement configuration in the context of sequential data assimilation methods [74,75]. Typically, inflation functions are regarded as functions of the singular values of background or analysis perturbations. However, some researches have demonstrated that it is more beneficial to view inflation functions as functions of the reduction factors of background singular values after assimilation [81]. The optimal configuration of the inflation factor can be studied in the future work.
(2) Smoother extension: The smoother extension of the LESTKF can be another improvement for the method we proposed. For example, the smoothing extension of the traditional ensemble filters effectively reduces the errors in the state estimates, compared to the filters [82,83,84].
(3) Machine learning: In recent years, machine learning methods have played a significant role in advancing the field of data assimilation. For instance, a new Hybrid Data Assimilation (DA) method based on a Machine Learning (HDA-ML) method overcomes the drawbacks of the traditional hybrid 4DVar-EnKF method by using neural networks to replace the tangent linear and adjoint models, and adopting a convolutional neural network (CNN) model to adaptively combine the results of 4DVar and EnKF [85]. He et al. [86] introduces a hybrid Data Assimilation and Machine Learning framework (DA-ML method) implemented in the Weather Research and Forecasting (WRF) model to optimize surface soil and vegetation conditions. The results demonstrated that the WRF (DA-ML) model effectively improves estimations of sensible and latent heat fluxes, evapotranspiration, air temperature, and specific humidity, reducing biases and simulating more realistic oasis–desert interactions. The machine learning will be integrated in our data assimilation framework in our next work.

## 5. Conclusions

This study serves as a groundbreaking exploration of the potential in situ soil moisture observations hold for improving regional soil moisture estimation. There are three main findings in this study: (1) The Local Error-Subspace Transform Kalman Filter (LESTKF) was effective in updating local model states, with improved spatial and temporal assimilation performance as the observation search radius increased. (2) Optimal data assimilation performance was achieved with an observation search radius exceeding 20 km, demonstrating the importance of careful selection of the range for in situ soil moisture observations. (3) Incorporating wireless sensor network data into the distributed model proved to be a significant departure from conventional approaches, highlighting the value of WATERNET’s high accuracy and resolution capabilities for regional soil moisture observation. Additionally, WATERNET’s ability to provide multiple-layered soil temperature and moisture observations opens new avenues for the integration of these variables into our data assimilation framework, further improving hydrological state estimations [87]. The study has significant implications for small-scale water resources research and management, particularly for freshwater resource scheduling (e.g., field scale irrigation) at small basin scales [88]. Future work will focus on joint assimilation of satellite soil moisture and in situ observations for multi-variable assimilation applications, optimization of the inflation factor, and machine learning methods within the chosen approach [89]. Additionally, leaf area index and streamflow data assimilation [18] will be considered, as well as coupling with WRF model extension.

## Figures and Tables

**Table 1:** Data characteristics and sources.

Name	Spatial Resolution	Sources
DEM	90 m	SRTM (https://www.earthdata.nasa.gov/sensors/srtm/ (accessed on 1 January 2023))
Soil type map	1 km	HWSD v1.2 (https://www.fao.org/soils-portal/data-hub/soil-maps-and-databases/harmonized-world-soil-database-v12/en/ (accessed on 1 January 2023))
Land use type	500 m	MCD12Q1 (https://ladsweb.modaps.eosdis.nasa.gov/missions-and-measurements/products/MCD12Q1 (accessed on 1 January 2023))
AMS	-	HiWATER (http://poles.tpdc.ac.cn/en/ (accessed on 1 January 2023))

**Table 2:** Descriptions of the calibrated parameters.

Parameter Name	Description	Level
SURLAG	Surface runoff lag time (days)	Basin
ESCO	Soil evaporation compensation factor	Basin
CH_K2	Effective hydraulic conductivity of the main channel alluvium (mm/h)	Subbasin
CH_N2	Manning’s “n” value for the main channel	Subbasin
ALPHA_BF	Base flow alpha factor (days)	Grid cell
CN2	Initial SCS-CN II value	Grid cell
SOL_AWC	Available water capacity (mm H_2_O/mm soil)	Grid cell
SOL_K	Saturated hydraulic conductivity (mm/h)	Grid cell
SFTMP	Snowfall temperature (°C)	Grid cell

**Table 3:** Statistics of improvement (RMSEop−RMSEda m^3^/m^3^) based on LESTKF with different observation search radiuses with observation standard deviation of 0.03 m^3^/m^3^.

Observation Standard Deviation Error		Observation Search Radiuses					
		0 km	5 km	10 km	20 km	40 km	50 km
0.01 m^3^/m^3^	Min	−0.012	−0.014	−0.021	−0.024	−0.024	−0.026
	Max	0.092	0.092	0.093	0.121	0.091	0.108
	Mean	0.002	0.003	0.004	0.006	0.006	0.0054
	Std	0.003	0.005	0.007	0.009	0.009	0.009
0.03 m^3^/m^3^	Min	−0.013	−0.016	−0.023	−0.026	−0.026	−0.028
	Max	0.09	0.09	0.091	0.119	0.089	0.106
	Mean	0	0.001	0.002	0.004	0.004	0.004
	Std	0.003	0.005	0.007	0.01	0.01	0.01
0.05 m^3^/m^3^	Min	−0.016	−0.019	−0.026	−0.029	−0.029	−0.031
	Max	0.077	0.086	0.086	0.114	0.083	0.102
	Mean	0	0.0002	0.001	0.001	0.001	0.0004
	Std	0.003	0.006	0.008	0.01	0.011	0.011

**Table 4:** The performance of soil moisture estimation in LESTKF and EnKF during open-loop periods.

	Observation Standard Error (m^3^/m^3^)	*Pbias* (%)	RMSE (m^3^/m^3^)	CPU Time (Second)	The Number of Soil Moisture Gauges
Open-loop		24.87	0.025	12.105	
LESTKF	0.01	20.29	0.019	19.105	32
	0.03	21.36	0.021	19.105	32
	0.05	22.43	0.024	19.105	32
EnKF	0.01	24.87	0.025	143.356	32
	0.03	24.87	0.025	143.356	32
	0.05	24.87	0.025	143.356	32

## Data Availability

Data are contained within the article.
